# High-speed and energy-efficient asynchronous carry look-ahead adder

**DOI:** 10.1371/journal.pone.0289569

**Published:** 2023-10-05

**Authors:** Padmanabhan Balasubramanian, Weichen Liu

**Affiliations:** School of Computer Science and Engineering, Nanyang Technological University, Singapore, Singapore; Universiti Teknologi Malaysia, MALAYSIA

## Abstract

Addition is a fundamental computer arithmetic operation that is widely performed in microprocessors, digital signal processors, and application-specific processors. The design of a high-speed and energy-efficient adder is thus useful and important for practical applications. In this context, this paper presents the designs of novel asynchronous carry look-ahead adders (CLAs) viz. a standard CLA (SCLA) and a block CLA (BCLA). The proposed CLAs are monotonic, dual-rail encoded, and are realized according to return-to-zero handshake (RZH) and return-to-one handshake (ROH) protocols using a 28-nm CMOS process technology. The proposed BCLA has a slight edge over the proposed SCLA, and the proposed BCLA reports the following optimizations in design metrics such as cycle time (delay), area, and power compared to a recently presented state-of-the-art asynchronous CLA for a 32-bit addition: (i) 32.6% reduction in cycle time, 29% reduction in area, 4.3% reduction in power, and 35.5% reduction in energy for RZH, and (ii) 31.4% reduction in cycle time, 28.9% reduction in area, 4.4% reduction in power, and 34.4% reduction in energy for ROH. Also, the proposed BCLA reports reductions in cycle time and power/energy compared to many other asynchronous adders.

## 1. Introduction

Asynchronous circuits, which utilize delay-insensitive codes [1] for data encoding and incorporate a four-phase handshake protocol for data communication, are called input-output (IO) mode asynchronous circuits, and they are more robust compared to synchronous circuits [2]. This is because IO-mode asynchronous circuits are not clock-driven unlike synchronous circuits, rather they are event-driven. This makes IO-mode asynchronous circuits naturally robust to process, voltage, and temperature variations [3, 4], and they are inherently elastic [5]. Besides, IO-mode asynchronous circuits are modular [6], and less affected by electromagnetic interference compared to synchronous circuits [7] and thus they are suited for secure applications [8, 9]. Further, IO-mode asynchronous circuits are self-checking [10].

IO-mode asynchronous circuits may or may not be delay-insensitive i.e., quasi-delay-insensitive (QDI) in practice. QDI circuits assume the presence of isochronic forks [11]. An isochronic fork refers to an electrical node from where two or more wires may emerge, and signal transitions (i.e., binary 0 to 1 or 1 to 0) on the wires are assumed to happen concurrently. The isochronic fork assumption is practically viable for microelectronics and nanoelectronics [12]. Quasi-delay-insensitivity implies that all the output(s) of a circuit is produced after all the inputs are received and the full completion of internal processing within the circuit. This makes QDI asynchronous circuits robust but at the expense of compromising on the implementation cost (i.e., area, power, and speed) compared to non-QDI asynchronous circuits. There exist different types of QDI circuits such as strong-indication circuits [13], weak-indication circuits [13], and early output circuits [14]. Strong indication circuits require all the primary inputs to process and produce all the primary outputs. Weak-indication circuits can process a subset of primary inputs to produce a subset of primary outputs; at the maximum, all but one of the primary outputs could be produced after processing a subset of primary inputs. But only after receiving the last primary input, a weak-indication circuit would complete the processing to produce the last primary output. Early output (QDI) circuits may process a subset of primary inputs received to produce all of the primary outputs.

On the other hand, IO-mode asynchronous circuits that are not QDI include relative-timed circuits [15] and monotonous/monotonic circuits [16, 17]. Relative-timed circuits tend to incorporate internal timing assumption(s) to sequence the input(s) to process and produce the output(s), whereas monotonic circuits are less complicated in that they only guarantee the monotonicity of signal transitions in a circuit. Monotonicity [16] means rising signal transitions (say, binary 0 to 1) on the inputs of a circuit would result in rising signal transitions on the outputs of a circuit, and falling signal transitions (say, binary 1 to 0) on the inputs of a circuit would result in falling signal transitions on the outputs of a circuit. A circuit may be monotonically increasing (i.e., monotonic for rising signal transitions alone) or monotonically decreasing (i.e., monotonic for falling signal transitions alone), monotonically increasing and decreasing (i.e., monotonic for both rising and falling signal transitions), or non-monotonic. Generally, synchronous circuits tend to be non-monotonic whereas IO-mode asynchronous circuits are monotonic. Henceforth, in this paper, by ‘monotonic circuits’, we mean both monotonically increasing and decreasing implementations of asynchronous circuits unless stated otherwise.

The different types of QDI circuits mentioned earlier viz. strong-indication, weak-indication, and early output are physically realized as monotonic implementations (i.e., both monotonically increasing and decreasing) but they are constrained to ensure the full completion of internal processing within a circuit before producing all the primary outputs. Relative-timed circuits are a kind of early output circuits, but they may incorporate sophisticated timing assumption(s) to sequence the processing of inputs to produce the outputs. Monotonic circuits tend to be early output circuits but they do not mandate the full completion of internal processing to produce all the primary outputs. Hence, compared to QDI asynchronous circuits, non-QDI asynchronous circuits are rather relaxed. This relaxation is practically viable and is welcome to reduce the complexity and implementation cost of an asynchronous circuit design and to make an asynchronous circuit feature superior performance metrics. The proposed asynchronous adders are monotonic circuits that are found to enable significant optimization in the design metrics compared to existing asynchronous adders.

While QDI circuits avoid wire and gate orphans, relative-timed circuits and monotonic circuits may not. A wire orphan refers to an unacknowledged signal transition on a wire and a gate orphan refers to an unacknowledged signal transition on a gate’s output. Wire and gate orphans have been explained using examples in [18], and an interested reader may refer to the same for details. The issue of wire orphans is overcome by imposing the isochronic assumption on primary input forks while the issue of gate orphans is dealt with by performing indicating logic decomposition in QDI circuits [19, 20]. Monotonic circuits, as they are non-QDI (internally), neglect the issue of gate orphans since monotonicity is guaranteed for both rising and falling signal transitions between the primary inputs and primary outputs. A monotonic implementation of asynchronous circuits would avoid a collision between data given the insertion of a spacer between two successive data, and the acknowledgment of the complete receipt of data and spacer via handshaking between input and output registers. QDI circuits also include a spacer between two successive data. To ensure that monotonic circuits are free from wire orphans, the isochronic assumption can be imposed on the primary input forks as done in QDI circuits to detect the complete arrival of primary inputs, where the primary inputs to the current circuit may be the primary outputs produced from a preceding circuit.

In the remainder of this paper, Section 2 discusses the preliminaries of asynchronous circuit design such as data encoding and four-phase handshaking. Section 3 surveys related literature on asynchronous adders. Section 4 presents the design of the proposed asynchronous adders. Section 5 reports the design metrics of different asynchronous adders including the proposed ones and makes a comparative evaluation. Section 6 concludes this paper.

## 2. Asynchronous circuit design–Background

**Fig 1:**
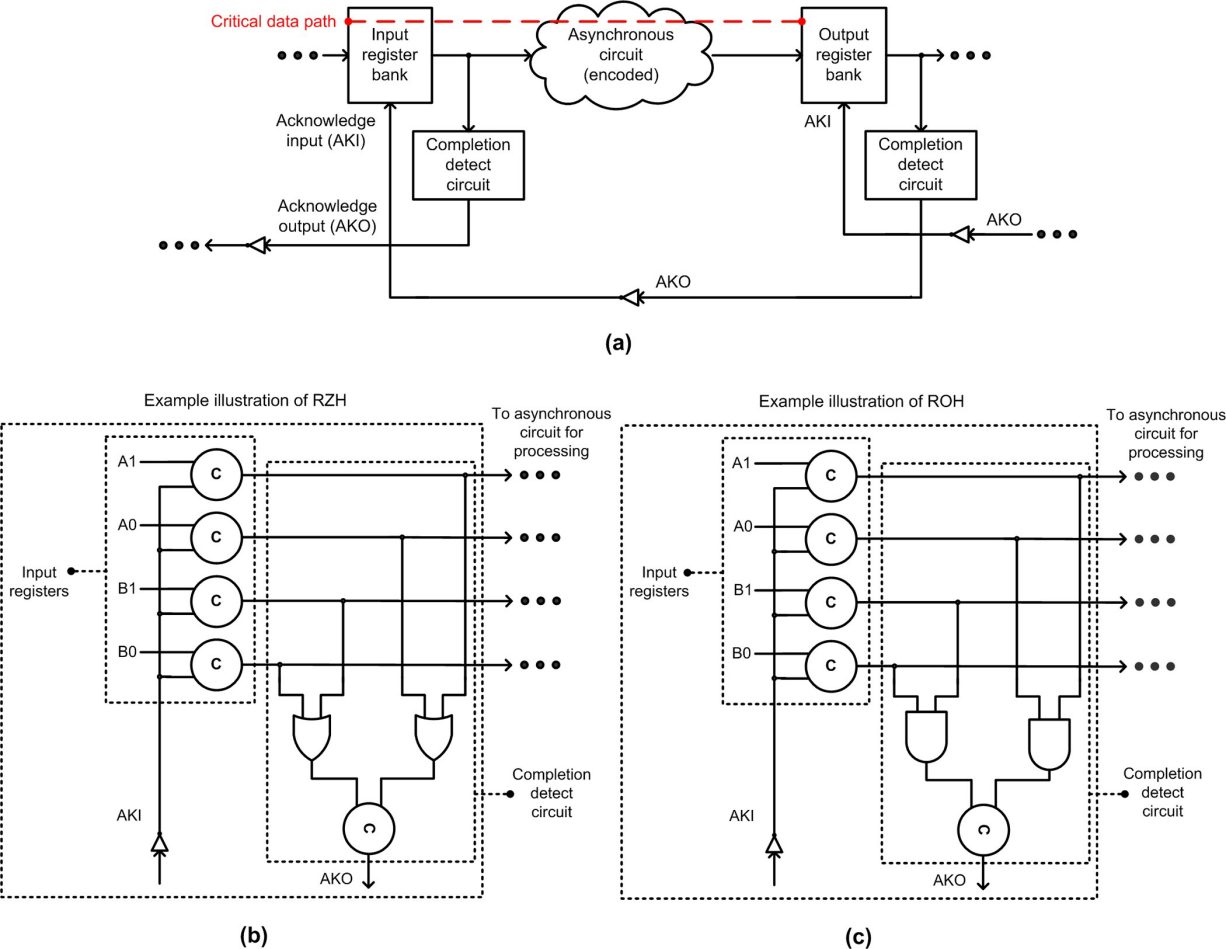
(a) Block diagram of an IO-mode asynchronous circuit stage. Example completion detect circuits corresponding to (b) RZH, (c) ROH, and input registers.

The block diagram of an IO-mode asynchronous pipeline stage is shown in Fig 1. An asynchronous circuit is sandwiched between two banks of registers viz. an input register bank, and an output register bank. The input register bank may serve as the output register bank for a preceding circuit in the pipeline, and the output register bank may serve as an input register bank for a succeeding circuit in the pipeline.

The asynchronous circuit receives input from the input registers, processes them, and produces the output which is sent to the output registers. The asynchronous circuit is encoded using a delay-insensitive code [1], and often the dual-rail code is used for data encoding–the encoding of data using dual-rail code shall be described later in this section. When all the outputs of an asynchronous circuit have reached the output registers, this is indicated (i.e., acknowledged) by a completion detect circuit, and the completion detect circuit associated with the output registers issues an acknowledgment output (AKO) signal which is then inverted to yield the acknowledgment input (AKI) signal that enables the input registers to forward new inputs to the asynchronous circuit for processing. Thus, AKO and AKI signals are Boolean complements. This communication process between the input and output register banks is called ‘handshaking’. There are four phases involved in handshaking in IO-mode asynchronous circuits, and there exist two types of handshake protocols: return-to-zero handshaking (RZH) and return-to-one handshaking (ROH). These shall also be described later in this section.

The Muller C-element [21] is used as a register in an IO-mode asynchronous circuit. The C-element is a unique gate, which outputs binary 1 when all its inputs are binary 1 and outputs binary 0 when all its inputs are binary 0. If the inputs to a C-element are not the same, the C-element shall retain its existing steady state. In the register banks, one of the inputs to each C-element is the AKI signal, and the other is an encoded input rail.

We shall now explain how data is encoded as per the handshaking scheme. Based on dual-rail encoding and RZH [2], an input I is represented using two wires or rails as say I1 and I0. I = 1 is encoded as I1 = 1 and I0 = 0, and I = 0 is encoded as I0 = 1 and I1 = 0. These two assignments are called ‘(valid) data’ with respect to RZH. I1 = I0 = 0 is referred to as the ‘spacer’ or ‘null data’, inserted between two successive data. I1 = I0 = 1 is an invalid/illegal assignment for RZH, which is avoided. Based on dual-rail encoding and ROH [22], an input I is represented using two wires or rails as say I1 and I0, where I = 1 is encoded as I1 = 0 and I0 = 1, and I = 0 is encoded as I0 = 0 and I1 = 1. These two assignments are called ‘(valid) data’ with respect to ROH. I1 = I0 = 1 is referred to as the ‘spacer’ or ‘null data’, inserted between two successive data. I1 = I0 = 0 for ROH is an invalid/illegal assignment, which is avoided.

Example illustrations of completion detect circuit corresponding to RZH and ROH are shown in Fig 1(B) and 1(C) respectively. For RZH, the completion detect circuit consists of a series of 2-input OR gates in the first level to combine the dual rails of respective encoded inputs. The outputs of OR gates are given to a C-element or a tree of C-elements which produce the AKO signal. For ROH, the completion detect circuit consists of a series of 2-input AND gates in the first level to combine the dual rails of respective encoded inputs. The outputs of AND gates are given to a C-element or a tree of C-elements which produce the AKO signal.

We shall now describe the four phases involved in RZH and ROH by referring to Fig 1(A). With respect to RZH, in the first phase, initially, AKI = 1 since AKO = 0, and the input register bank would forward the data for processing to the asynchronous circuit. This implies that one of the rails of each encoded input would be driven to binary 1, signifying the application of data for processing by the asynchronous circuit. In the second phase, the output register bank would receive all the outputs produced by the asynchronous circuit and the AKO signal of binary 1 is issued. In the third phase, the input register bank waits for AKI to become 0, and then the spacer (all zeroes) is supplied to the asynchronous circuit for processing. In the fourth and final phase, the output register bank receives the spacer output produced by the asynchronous circuit, and the AKO signal of binary 0 is issued which implies that AKI would subsequently assume binary 1 which signifies the completion of one data transaction.

With respect to ROH, in the first phase, initially, AKI = 1 since AKO = 0, and the input register bank would forward the spacer (all ones) for processing to the asynchronous circuit. This implies that all the rails of each encoded input would be driven to binary 1, signifying the application of the spacer for processing by the asynchronous circuit. In the second phase, the output register bank would receive the spacer output produced by the asynchronous circuit and the AKO signal of binary 1 is issued. In the third phase, the input register bank waits for AKI to become 0, and then data (where one of the rails of each encoded input is driven to binary 0) is supplied to the asynchronous circuit for processing. In the fourth and final phase, the output register bank receives the data output produced by the asynchronous circuit, and then the AKO signal of binary 0 is issued which implies that AKI would subsequently assume binary 1, which signifies the completion of one data transaction.

As explained in the handshaking process, a spacer is inserted between the application of two input data to ensure delay insensitivity in QDI circuits. For a monotonic circuit, the insertion of a spacer between two successive data helps to ensure delay insensitivity (externally) for handshaking. In a synchronous circuit, a pair of input and output registers would be controlled by a common clock signal, and the minimum clock period determines the maximum frequency of operation. The minimum clock period would be roughly calculated as the sum of set-up time, input register delay, and maximum combinational logic delay (also called critical path delay which is the main timing parameter) in a synchronous circuit. In an IO-mode asynchronous circuit that is shown in Fig 1, the ‘cycle time’ is the dominant timing parameter that represents the time taken for one data transaction. The cycle time is calculated as the sum of the times taken to process data and the spacer. The (worst-case) time taken to process data is called forward latency, and the (worst-case) time taken to process the spacer is called reverse latency. The cycle time of an IO-mode asynchronous circuit is given by the sum of forward and reverse latencies. The critical data path traversed in an IO-mode asynchronous circuit includes an input register bank and an asynchronous circuit, which is highlighted by the red dashed line in Fig 1(A). The critical data path delay encountered for processing data and spacer may differ in an IO-mode asynchronous circuit depending upon its type. This shall be discussed in the next section while surveying different asynchronous adders.

## 3. Survey of (IO-mode) asynchronous adders

The addition is a fundamental computer arithmetic operation that is frequently performed in microprocessors, digital signal processors, and application-specific processors such as graphics processing units, etc. Given this, the realization of addition using a high-speed and low-power/energy-efficient adder has practical significance. Several IO-mode asynchronous adders have been presented in the literature, many of which have been realized at the gate level while a few of them have been realized at the transistor level. Gate-level designs (also referred to as semi-custom designs) are relatively easier to implement/replicate compared to transistor-level designs (also referred to as full-custom designs) since the former uses the gates available in a standard cell library while the latter involves building circuits transistor-by-transistor and would require optimum sizing of transistor aspect ratios to achieve an acceptable trade-off between different performance metrics and to ensure that suitable driving strengths are provisioned at the cell level and the circuit level, depending upon the process technology used for implementation. Therefore, considering the design complexities involved, semi-custom design is preferable to full-custom design, and this holds well for asynchronous circuits.

The IO-mode asynchronous adders presented in the literature correspond to QDI and non-QDI design styles, and these are surveyed next with theoretical modeling of their timing performance. Note that the theoretical modeling of the cycle time of different asynchronous adders is approximate since only the delays of building blocks are topologically considered for simplicity and no gate, interconnect, or parasitic delays are considered. Besides, the input register delay is also not accounted for in the theoretical computation. The area of various asynchronous adders tends to differ depending upon their logic composition, and accordingly, their power dissipation also differs, and these metrics are non-trivial to model theoretically. The power dissipation of various IO-mode asynchronous adders does not vary significantly and this is due to the activation of unique signal paths from primary inputs to primary outputs for the processing of data and spacer in IO-mode asynchronous circuits and hence non-necessary signal transitions that do not reach the primary outputs generally do not occur. As a result, the switching activity and dynamic power do not vary significantly between different IO-mode asynchronous adders.

In synchronous design, the ripple-carry adder (RCA) is very slow although it occupies less area and dissipates less power than other high-speed adders such as a carry look-ahead adder (CLA). However, in IO-mode asynchronous design, the RCA architecture could be useful as some of the RCAs tend to have a small reverse latency, which is unlikely to be achieved by any other adder architecture. Moreover, the RCA has the least area occupancy compared to other adders. We shall survey IO-mode asynchronous adders next.

**Fig 2:**
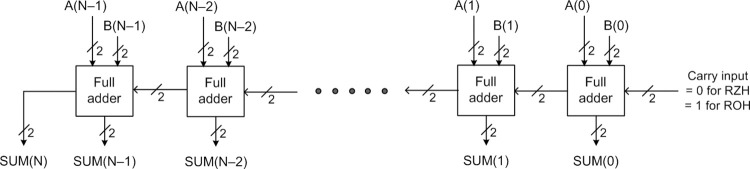
Dual-rail encoded asynchronous N-bit RCA comprising N full adders.

An N-bit RCA can be constructed by cascading N full adders, as shown in Fig 2, where A(N–1) up to A(0) and B(N–1) up to B(0) represent the inputs and SUM(N) up to SUM(0) represents the output. In Fig 2, the inputs and outputs of all the full adders are dual-rail encoded. A full adder adds two input bits along with a carry input and produces the sum output and any carry overflow. For example, the output equations of a dual-rail encoded full adder corresponding to RZH are given below, where (A1, A0) and (B1, B0) represent the dual-rail augend and addend, (C1, C0) represent the dual-rail carry input, (SM1, SM0) represents the dual-rail sum output, and (CT1, CT0) represents the dual-rail carry overflow from the addition.


SM1=A0B0C1+A0B1C0+A1B0C0+A1B1C1 (1)

SM0=A0B0C0+A0B1C1+A1B0C1+A1B1C0 (2)

CT1=A0B1C1+A1B0C1+A1B1C0+A1B1C1 (3)

CT0=A0B0C0+A0B0C1+A0B1C0+A1B0C0 (4)

The full adders based on [23, 24] are strongly indicating and a strong-indication full adder can be designed based on the delay-insensitive minterm synthesis (DIMS) method [25]. These full adders can be replicated and cascaded to form RCAs, which would have a forward latency of O[N×D_FA_] and a reverse latency of O[N×D_FA_], where N refers to the number of full adders used commensurate with the size of the addition. D_FA_ represents the propagation delay of a full adder. It should be noted that D_FA_ tends to differ for various full adders depending upon their logic composition. The forward and reverse latencies of an RCA incorporating strong-indication full adders are the highest, and this is because of the longest carry propagation encountered for the processing of both data and the spacer. Thus, the cycle time of such RCAs would be equal to O[2×N×D_FA_], which implies those RCAs would be very slow.

Reference [26] presented a weak-indication full adder, considered the ultimate full-custom design requiring 42 transistors for a static CMOS implementation. This full adder when replicated and cascaded to construct an RCA would have a forward latency of O[N×D_FA_] and a reverse latency of O[2×D_FA_], thus resulting in a cycle time of O[(N+2) ×D_FA_]. The forward latency is still significant and this is because of the maximum carry propagation encountered for the processing of data. However, the reverse latency is considerably minimized due to the distribution of weak indication between the sum and carry outputs of the full adder. The carry output of the full adder is dependent only upon the adder inputs for the processing of the spacer while the sum output of the full adder is dependent only upon the carry input for the processing of the spacer. As a result, the RCA can produce the spacer sum output with just two full adder delays.

Full adders realized based on [27‒29] are weakly indicating. A weak-indication full adder can also be realized based on the DIMS method [25]. The weak-indication full adders of [25, 27] have a cycle time of O[2×N×D_FA_] since their forward and reverse latencies are O[N×D_FA_], and this is due to the worst-case carry propagation encountered for the processing of both data and the spacer. The weak-indication full adders of [28, 29] have a cycle time of O[(N+2) ×D_FA_] given that their forward latency is O[N×D_FA_], and their reverse latency is O[2×D_FA_]. The reduction in reverse latency results from biased weak indication whereby the sum output of the full adders is made responsible for indicating all the adder inputs while the carry output is freed from indication.

Recently, [30] presented three weak-indication full-adder designs based on the concept of sorting networks (SN), namely SN full adder, SNFC full adder, and SNX full adder. However, these full adders were not physically implemented. Among these, SN full adder has a cycle time of O[2×N×D_FA_] implying that its forward and reverse latencies are the same. SNFC and SNX full adders have a reduced cycle time of O[(N+2) ×D_FA_] which implies that their reverse latency is significantly lesser than their forward latency due to the phenomenon of biased weak indication.

An early output QDI full adder was presented in [31], which when duplicated and cascaded to form an RCA would have a forward latency of O[N×D_FA_], and a reverse latency of O[2×D_FA_], which results in a cycle time of O[(N+2) ×D_FA_]. The RCA also corresponds to the early output type.

Two early output full adders were presented in [32], which can be individually duplicated and cascaded to form relative-timed RCAs. Among the early output full adders, one of them is better optimized for the area while the other is better optimized for latency. The RCAs would have a forward latency of O[N×D_FA_], and an optimal reverse latency of O[D_FA_] which becomes possible since all the full adders in the RCA can simultaneously produce the spacer as the sum output without having to wait for the spacer carry input. The resultant cycle time of relative-timed RCAs is O[(N+1) ×D_FA_].

It should be noted that the D_FA_ of all the full adders is not the same as this depends upon the internal logic of the respective full adders. From the above discussion, it may be noted that some of the RCAs have a small reverse latency of O[2×D_FA_] or O[D_FA_], which is unlikely to be achieved by any other adder. However, all the RCAs have a forward latency of O[N×D_FA_], which is substantial. To reduce the significant forward latency encountered in an RCA, a CLA may be used and this is discussed next.

Reference [33] presented a hierarchical QDI CLA with speed-up circuity called DICLASP, which is a full-custom transistor-level design requiring a total of (66×N– 4) transistors to realize an N-bit DICLASP. However, in [33], DICLASP was simulated only in a topological sense without any physical implementation; hence, no physical design metrics were estimated. References [34–38] presented many gate-level designs of QDI CLAs based on standard and block CLA architectures, which are easy to reproduce and modular, and they were physically realized and their design metrics were estimated. We shall review these gate-level asynchronous CLAs given that the proposed asynchronous CLAs are also gate-level designs.

**Fig 3:**
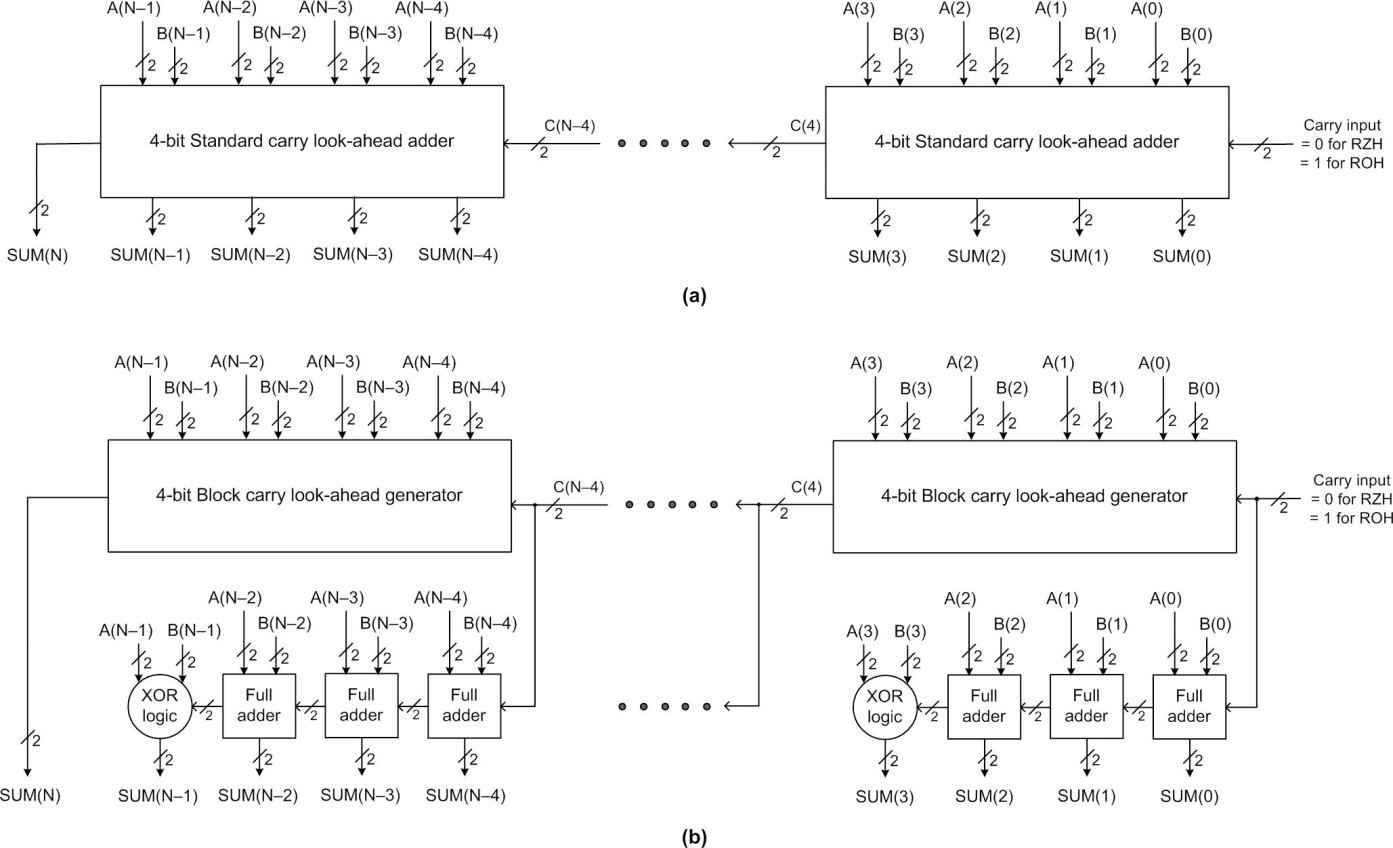
(a) N-bit standard CLA (SCLA), and (b) N-bit block CLA (BCLA). In the figure, N-bit SCLA and BCLA are constructed by replicating and cascading 4-bit SCLA and BCLA modules respectively for an example.

Two basic types of CLA architecture are available in the literature [39], namely the standard CLA (SCLA) and the block CLA (BCLA), which are illustrated by Fig 3(A) and 3(B) respectively. In Fig 3, A(N–1) up to A(0) represents one of the inputs and B(N–1) up to B(0) represents the other input given to the CLAs. SUM(N) up to SUM(0) represents the sum output of the addition, where SUM(N) represents any carry overflow resulting from the addition. In the inputs, A(N–1) and B(N–1) are the most significant, and A(0) and B(0) are the least significant. In the output, SUM(N) is the most significant, and SUM(0) is the least significant. Note that the inputs and outputs of the CLAs are dual-rail encoded according to the handshake protocol used. In general, (N/M) M-bit CLA modules can be used to construct an N-bit CLA where N and M are even and N modulo M equals 0. In Fig 3(A) and 3(B), M is assumed to be 4. We shall first discuss the SCLA architecture followed by the BCLA architecture.

An M-bit SCLA module receives 2M inputs with/without any carry input and processes them to produce M sum outputs and a look-ahead carry output, which in turn serves as the carry input for a successive SCLA module. In single-rail format, the basic equation for the carry output from addition is given by (5), where A_Q_ and B_Q_ represent the augend and addend corresponding to a Qth adder stage, C_Q_ represents the carry input, and C_Q+1_ represents the carry output.

$$C_{Q+1}=A_{Q}B_{Q}+(A_{Q}⊕B_{Q})C_{Q}=G_{Q}+P_{Q}C_{Q}$$ (5)

Eq (5) can be utilized to derive a look-ahead carry output for a CLA module through recursion. The product term A_Q_B_Q_ is referred to as the ‘carry generate function’ and the term (A_Q_ ⊕ B_Q_) is referred to as the ‘carry propagate function’. A carry output may be generated from a Qth adder stage based on the activation of the generate function, and the carry input to a Qth adder stage may be forwarded as the carry output based on the activation of the propagate function.

Based on the dual-rail encoding and RZH, A_Q_, B_Q,_ and C_Q_ are encoded as (A^1^_Q_, A^0^_Q_), (B^1^_Q_, B^0^_Q_), and (C^1^_Q_, C^0^_Q_) respectively. The equations for the carry output’s dual rails are given by (6) and (7). In (7), K_Q_ denotes the ‘carry kill function’, which implies that the carry input to a Qth adder stage is killed, so no carry output is produced (i.e., the carry output is 0). The generalized dual-rail encoded expressions for carry propagate, generate, and kill functions are given as G_Q_ = A^1^_Q_B^1^_Q_; P_Q_ = A^1^_Q_B^0^_Q_ + A^0^_Q_B^1^_Q_; and K_Q_ = A^0^_Q_B^0^_Q_.

$$C^{1}{}_{Q+1}=G_{Q}+P_{Q}C^{1}{}_{Q}$$ (6)

$$C^{0}{}_{Q+1}=K_{Q}+P_{Q}C^{0}{}_{Q}$$ (7)

In an SCLA, in every M-bit SCLA module, M look-ahead carry outputs are generated in parallel out of which (M– 1) carry outputs are used to produce the respective sum bits belonging to that SCLA module and the most significant carry output is given as the carry input to a successive SCLA module. However, the look-ahead carry output generated by the last (i.e., most significant) SCLA module signifies the carry overflow from the addition which is represented by SUM(N).

Given a QDI implementation of an N-bit SCLA [34], which is shown in Fig 3(A), its forward latency and reverse latency would be the same, that is governed by O[D_SCLA_^4b_first^ + {(N/M)– 1}×D_SCLA_^4b^], where D_SCLA_^4b_first^ denotes the propagation delay encountered in the first 4-bit SCLA module that processes inputs A(3) to A(0) and B(3) to B(0), and D_SCLA_^4b^ denotes the propagation delay encountered in each subsequent 4-bit SCLA module that extends up to the last SCLA module. D_SCLA_^4b_first^ is distinguished from D_SCLA_^4b^ in that the look-ahead carry C(4) output by the first 4-bit SCLA module is produced after traversing multiple levels of logic whereas the look-ahead carry output by successive 4-bit SCLA modules such as C(8), C(12), etc. are produced after traversing relatively reduced levels of logic. Therefore, the cycle time of an N-bit SCLA is given by O [{D_SCLA_^4b_first^ + ((N/M)– 1)×D_SCLA_^4b^)} ×2]. The same order of cycle time would be applicable for N-bit SCLAs realized using strong-indication and weak-indication logic synthesis methods [25–27]. However, the logic composition of the SCLA given in [34] is more optimized compared to the logic composition of the SCLA realized using [25–27], and hence the latter is not preferable.

Fig 3(B) shows an N-bit BCLA constructed using (N/4) 4-bit BCLA modules. Each 4-bit BCLA module consists of a 4-bit block carry look-ahead generator (BCLG), 3 full adders, and a 3-input XOR function. Unlike an SCLA, in a BCLA only one look-ahead carry output is produced by each M-bit BCLA module which serves as the carry input for a successive BCLA module. However, the look-ahead carry output generated by the last (i.e., most significant) BCLA module signifies the carry overflow from the addition which is represented by SUM(N). Each M-bit BCLA module internally consists of an M-bit RCA to process and produce M sum bits corresponding to that BCLA module and the carry overflow from the M-bit RCA is discarded. Hence, instead of a full adder a 3-input XOR function would suffice to produce the most significant sum output of each BCLA module.

Given a QDI implementation of an N-bit BCLA [35–38], shown in Fig 3(B), its forward latency is governed by O[D_BCLG_^4b_first^ + {(N/M)– 2}×D_BCLG_^4b_intermediate^ + D_RCA_^4b^], and its reverse latency is governed by O[D_BCLG_^4b_first^ + {(N/M)– 2}×D_BCLG_^4b_intermediate^ + D_FA_]. Here, D_BCLG_^4b_first^ signifies the propagation delay encountered in the first 4-bit BCLA module that processes inputs A(3) to A(0) and B(3) to B(0). D_BCLG_^4b_intermediate^ denotes the propagation delay encountered in every subsequent 4-bit BCLA module up to the penultimate BCLA module. D_BCLG_^4b_first^ is distinguished from D_BCLG_^4b_intermediate^ in that the look-ahead carry C(4) output by the first 4-bit BCLA module is produced after traversing multiple levels of logic whereas the look-ahead carry output by successive 4-bit BCLA modules such as C(8), C(12), etc. are produced after traversing relatively reduced levels of logic. D_RCA_^4b^ denotes the propagation delay encountered in the 4-bit RCA present in the last 4-bit BCLA module. D_FA_ denotes the propagation delay of a full adder. The forward latency and reverse latency of an N-bit BCLA are close, and its cycle time is given by O[{D_BCLG_^4b_first^ + ((N/M)– 2) ×D_BCLG_^4b_intermediate^)} ×2 + D_RCA_^4b^ + D_FA_]. An N-bit BCLA constructed using strong-indication and weak-indication logic synthesis methods of [25–27] would have a cycle time expressed by O[{D_BCLG_^4b_first^ + ((N/M)– 2)×D_BCLG_^4b_intermediate^) + D_RCA_^4b^} ×2], which is somewhat greater than the above-mentioned cycle time for an N-bit BCLA utilizing early output logic. Moreover, [25–27] involves more logic and dissipates more power, as observed in [35], which is not preferable.

**Fig 4:**
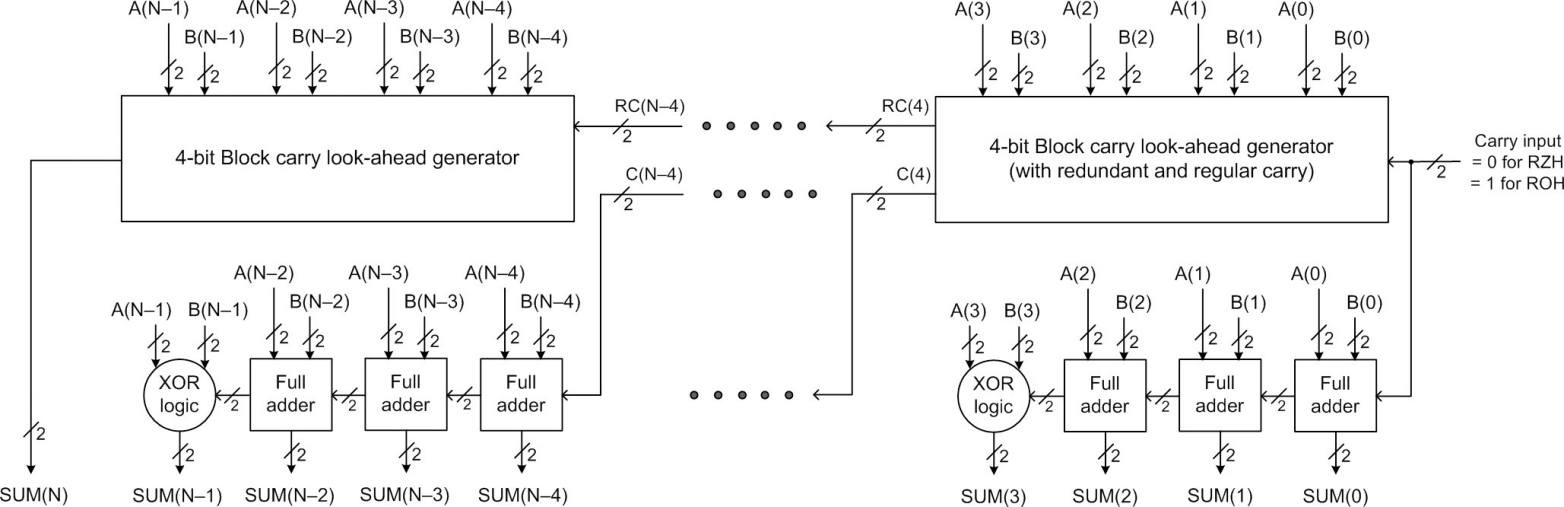
Example illustration of an N-bit BCLA comprising double carry logic (BCLADC).

The cycle times of N-bit QDI SCLA and BCLA mentioned above are rather significant and likely to exceed the cycle time of certain N-bit QDI RCAs, as noted in [37, 38]. Hence, a novel variant of the BCLA architecture that contains double carry logic was presented in [35], which is especially suited for IO-mode asynchronous design–we shall refer to this as the BCLADC architecture here. Improved versions of the BCLADC were subsequently presented in [36–38]. The architecture of an N-bit BCLADC is shown in Fig 4 for an illustration.

In contrast to an N-bit BCLA, shown in Fig 3(B), an N-bit BCLADC is constructed using [(N/M)– 1] BCLADC modules and a most significant BCLA module. Similar to Fig 3, in Fig 4, N and M are assumed to be even; N modulo M equates to 0; and M = 4. While an M-bit BCLA module consists of an M-bit BCLG and an M-bit RCA, as seen in Fig 3(B), an M-bit BCLADC module consists of an M-bit BCLG with double carry logic (i.e., M-bit BCLGDC) and an M-bit RCA as seen in Fig 4.

A BCLADC module produces two logically equivalent look-ahead carry outputs called ‘regular’ and ‘redundant’ carry outputs, which are denoted by the notations ‘C’ and ‘RC’ in Fig 4 respectively. The redundant look-ahead carry output generated by a BCLADC module is given as the carry input for the BCLG present in the successive BCLADC or BCLA module. On the other hand, the regular look-ahead carry output generated by a BCLADC module is given as the carry input to the RCA present in the successive BCLADC or BCLA module. In a BCLADC module, the redundant look-ahead carry output is generated relatively faster than the regular look-ahead carry output. This is because the redundant look-ahead carry output is freed from the indication constraint thereby paving the way for an early generation while the regular look-ahead carry output is made responsible for indication. The carry signal is thus propagated faster between the BCLADC modules due to the redundant carry logic, and this helps to improve the speed of a BCLADC. This speed improvement comes at the expense of just moderate increases in area and power for a BCLADC compared to a BCLA due to the doubling of the carry logic.

Concerning Figs 3 and 4, although strong-indication or weak-indication or early output timing models may be used for realizing SCLA, BCLA, and BCLADC modules, an early output realization was found to be preferable to achieve high speed and good energy efficiency, as observed in [37, 38]. Among the different IO-mode asynchronous CLAs [34–38], the BCLADC presented in [38] was found to be of higher speed (i.e., lesser cycle time) and more energy-optimized than its counterparts. The forward latency of an N-bit BCLADC, as per the architecture shown in Fig 4, is O[D_BCLGDC_^4b_first^ + {(N/M)– 2}×D_BCLGDC_^4b_intermediate^ + D_RCA_^4b^], and the reverse latency is observed to be O[D_BCLGDC_^4b_first^ + D_BCLGDC_^4b_intermediate^ + D_FA_] [37, 38]. D_BCLGDC_^4b_first^ refers to the delay encountered in the first 4-bit BCLGDC module that processes inputs A(3) to A(0) and B(3) to B(0), D_BCLGDC_^4b_intermediate^ refers to the delay encountered in an intermediate 4-bit BCLGDC module, D_RCA_^4b^ refers to the delay encountered in the 4-bit RCA of the last 4-bit BCLA module that processes inputs A(N–1) to A(N–3) and B(N–1) to B(N–3). Thus, the cycle time of the N-bit BCLADC would be O[2× D_BCLGDC_^4b_first^ + {(N/M)– 1}×D_BCLGDC_^4b_intermediate^ + D_RCA_^4b^ + D_FA_], which is less than the cycle time of N-bit SCLA and BCLA counterparts.

Although there exist other adder architectures such as the carry select adder (CSLA), and parallel-prefix adders (PFAs) in the literature, which are high-speed for synchronous design, concerning IO-mode asynchronous design they may not be high-speed due to the consideration of reverse latency and cycle time which are not accounted for in the synchronous design. As mentioned earlier, in a synchronous design, the critical path delay is the main timing parameter which is equivalent to the forward latency of an IO-mode asynchronous design. IO-mode asynchronous CSLAs comprising uniform and non-uniform input partitions were realized in [40], but as noted in [38], a 32-bit asynchronous CSLA featuring a uniform input partition where the inputs are split into 4 groups of 8-bit each has a reverse latency that is 77% (78%) of the forward latency for RZH (ROH), and a 32-bit asynchronous CSLA featuring an optimum non-uniform input partition where the inputs are split into 7 groups containing 8-, 7-, 6-, 4-, 3-, 2-, and 2- bits has almost the same forward and reverse latencies for RZH and ROH. Among these, the asynchronous CSLA featuring a uniform input partition was found to have lesser forward and reverse latencies and thus less cycle time than its counterpart featuring a non-uniform input partition. Nevertheless, the asynchronous CSLA of [40] featuring a uniform input partition reported increased latencies and cycle time, and increased area and power dissipation compared to the asynchronous BCLADC of [37, 38], and increased latencies and cycle time, and increased area and power dissipation compared to many asynchronous RCAs [28, 29, 31, 32]. Hence, IO-mode asynchronous CLA is preferable to IO-mode asynchronous CSLA. Further, to our knowledge, no efficient IO-mode asynchronous PPA has been presented in the literature. In the next section, we present the design of monotonic asynchronous CLAs that is found to surpass the best existing asynchronous BCLADC in terms of all the design metrics.

## 4. Proposed asynchronous CLAs

We present novel monotonic (non-QDI) asynchronous CLAs which, as per definition, guarantee the monotonic relationship between the primary adder inputs and adder outputs. The indication requirement is relaxed and the full completion of internal processing within the circuit (especially for processing the spacer) is not mandated. The arrival of circuit inputs is acknowledged by the completion detect circuit that is associated with the input register bank. Given these, the use of the BCLADC architecture involving a double carry logic is not necessary; rather, the SCLA and BCLA architectures would be sufficient to ensure monotonicity. Therefore, monotonic N-bit SCLA and N-bit BCLA were realized using the proposed monotonic SCLA and BCLA modules following the architectures shown in Fig 3(A) and 3(B).

It was stated in the previous section that the QDI SCLA and BCLA architectures have high forward and reverse latencies, and this results in a high cycle time therefore they are inferior to a QDI BCLADC. However, the proposed SCLA and BCLA being monotonic have a reduced forward latency and a substantially reduced reverse latency in comparison, and hence the cycle time of the proposed SCLA and BCLA was found to be lesser than the cycle time of the QDI BCLADC (the results-based evidence for this shall be presented in the next section). This is because the logical composition of the proposed SCLA and BCLA modules differs from the conventional SCLA and BCLA modules, and these are discussed next. The physical realizations of existing SCLA, BCLA, and BCLADC are shown in [34–38], and an interested reader may refer to the same for details, so they are not repeated here.

**Fig 5:**
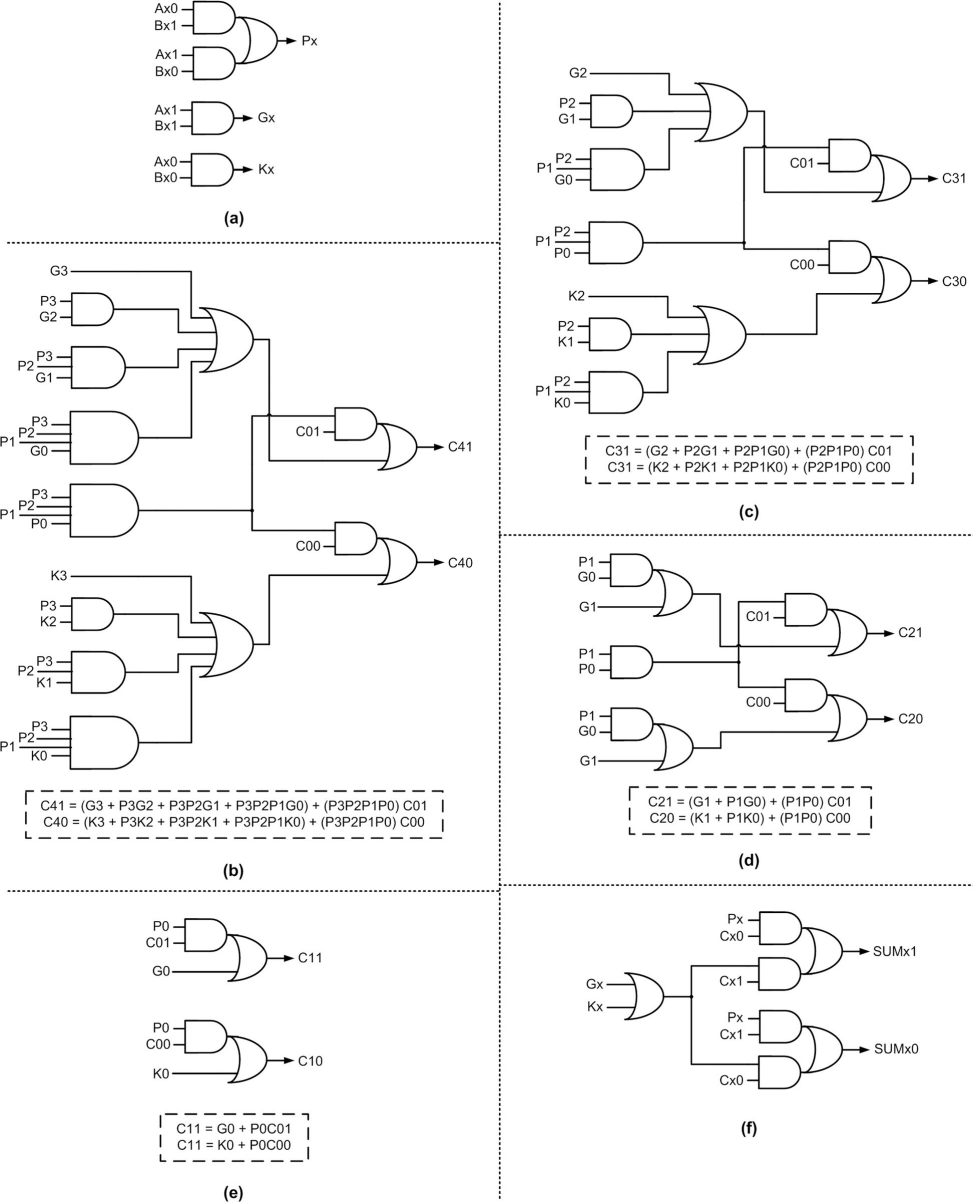
Building blocks of proposed monotonic asynchronous SCLA based on dual-rail encoding, corresponding to RZH: (a) generic realization of carry propagate, generate, and kill functions; (b)–(e) example implementation of 4-bit look-ahead carry outputs (C41, C40) up to (C11, C10); and (f) generic realization of sum output.

Fig 5 shows the logical realization of the proposed monotonic SCLA module, which employs dual-rail encoding and corresponds to RZH. To obtain the equivalent SCLA module corresponding to ROH, all the gates shown in Fig 5 should be replaced by their duals–this transformation principle [41] has already been proven in [42]. For example, AND, OR, AO21, and AO22 gates in Fig 5 should be replaced by OR, AND, OA21, and OA22 gates respectively to obtain the equivalent circuit corresponding to ROH. The monotonic SCLA module shown in Fig 5 is 4 bits in size, and this can be used to realize an N-bit monotonic SCLA as per the schematic shown in Fig 3(A). Nevertheless, any M-bit SCLA module can be realized by taking a cue from Fig 5.

In Fig 5A and 5B represent 4-bit inputs, (C01, C00) represents the carry input. (C41, C40) up to (C11, C10) represent the look-ahead carry outputs, and (SUMx1, SUMx0) represents an arbitrary sum output. P3 to P0, G3 to G0, and K3 to K0 represent the carry propagate, generate, and kill signals respectively. Fig 5(A) shows the generic realization of carry propagate, carry generate, and carry kill functions, and the notation ‘x’ denotes a bit position. Fig 5(B) to 5(E) show the realization of look-ahead carry outputs (C41, C40), (C31, C30), (C21, C20), and (C11, C10) along with their respective equations given within the dashed rectangle boxes. Fig 5(F) shows the realization of an arbitrary sum output.

From Fig 5(B) to 5(E), it may be noted that the carry input (C01, C00) is directly related to a look-ahead carry output through a single complex gate i.e., AO21 gate. This would help to achieve an optimal propagation delay in each intermediate SCLA module present in an N-bit SCLA. From (6) and (7), it may be observed that the carry output (C^1^_Q+1_, C^0^_Q+1_) can be physically related to the carry input (C^1^_Q_, C^0^_Q_) using a single complex gate viz. an AO21 gate. The same observation has been used to modify the logic expressions of all the look-ahead carry outputs (C41, C40) up to (C11, C10) such that these are physically related to the carry input (C01, C00) using individual AO21 gates. For example, referring to Fig 5(D), the logic expressions of (C21, C20) are initially given as,

C21=G1+P1G0+P1P0C01 (8)

C20=K1+P1K0+P1P0C00 (9)

We now introduce some intermediate Boolean variables say Z1, Z2, Z3, Z4, and Z5, and make the following assignments: Z1 = P1G0; Z2 = P1K0; Z3 = P1P0; Z4 = G1 + Z1; and Z5 = K1 + Z2, which result in simplified expressions for C21 and C20, given by (10) and (11). Subsequently, (10) and (11) can be realized using two individual AO21 gates, as seen in Fig 5(D).

C21=Z4+Z3C01 (10)

C20=Z5+Z3C00 (11)

This method of assigning intermediate Boolean variables to simplify the logic expressions of look-ahead carry outputs would help to achieve optimal carry propagation delay (i.e., one AO21 gate delay) in each intermediate SCLA module that would be incorporated in an N-bit SCLA. Eventually, the forward latency of the N-bit SCLA would be optimized. When the 4-bit SCLA module, shown in Fig 5, is replicated and cascaded to form an N-bit SCLA, its forward latency would be given by O[D_SCLA_^4b_first^ + {(N/M)– 2}×D_SCLA_^4b_intermediate^ + D_SCLA_^4b_last^], where D_SCLA_^4b_first^ denotes the propagation delay encountered in the first 4-bit SCLA module that processes inputs A(3) to A(0) and B(3) to B(0) in Fig 3(A), D_SCLA_^4b_intermediate^ denotes the propagation delay encountered in each successive 4-bit SCLA module excepting the last 4-bit SCLA module, and D_SCLA_^4b_last^ denotes the propagation delay encountered in the last 4-bit SCLA module. There would only be a slight difference in delay between D_SCLA_^4b_intermediate^ and D_SCLA_^4b_last^ given that the former relates to a most significant look-ahead carry output generation involving an AO21 gate while the latter relates to the production of the most significant adder sum bit using an AO22 gate, as seen from Fig 5. D_SCLA_^4b_first^ is distinguished from D_SCLA_^4b_intermediate^ in that the look-ahead carry C(4) output by the first 4-bit SCLA module is produced after traversing multiple levels of logic whereas the look-ahead carry output by successive 4-bit SCLA modules is produced after traversing one level of logic (i.e., an AO21 gate). Contrary to the N-bit QDI SCLA, the reverse latency of the N-bit monotonic SCLA is governed by an optimal O[D_SCLA_^4b^], which would be approximately equal to O[D_SCLA_^4b_first^]. The reduction in reverse latency becomes feasible since all the SCLA modules comprising the proposed SCLA can process and output the spacer in parallel without waiting for the spacer carry’s arrival due to the monotonic logic realization. The cycle time of the proposed N-bit monotonic SCLA is O[(D_SCLA_^4b_first^ ×2) + {(N/M)– 2}×D_SCLA_^4b_intermediate^ + D_SCLA_^4b_last^], which is substantially less than the cycle times of QDI SCLA, BCLA, and BCLADC discussed earlier.

**Fig 6:**
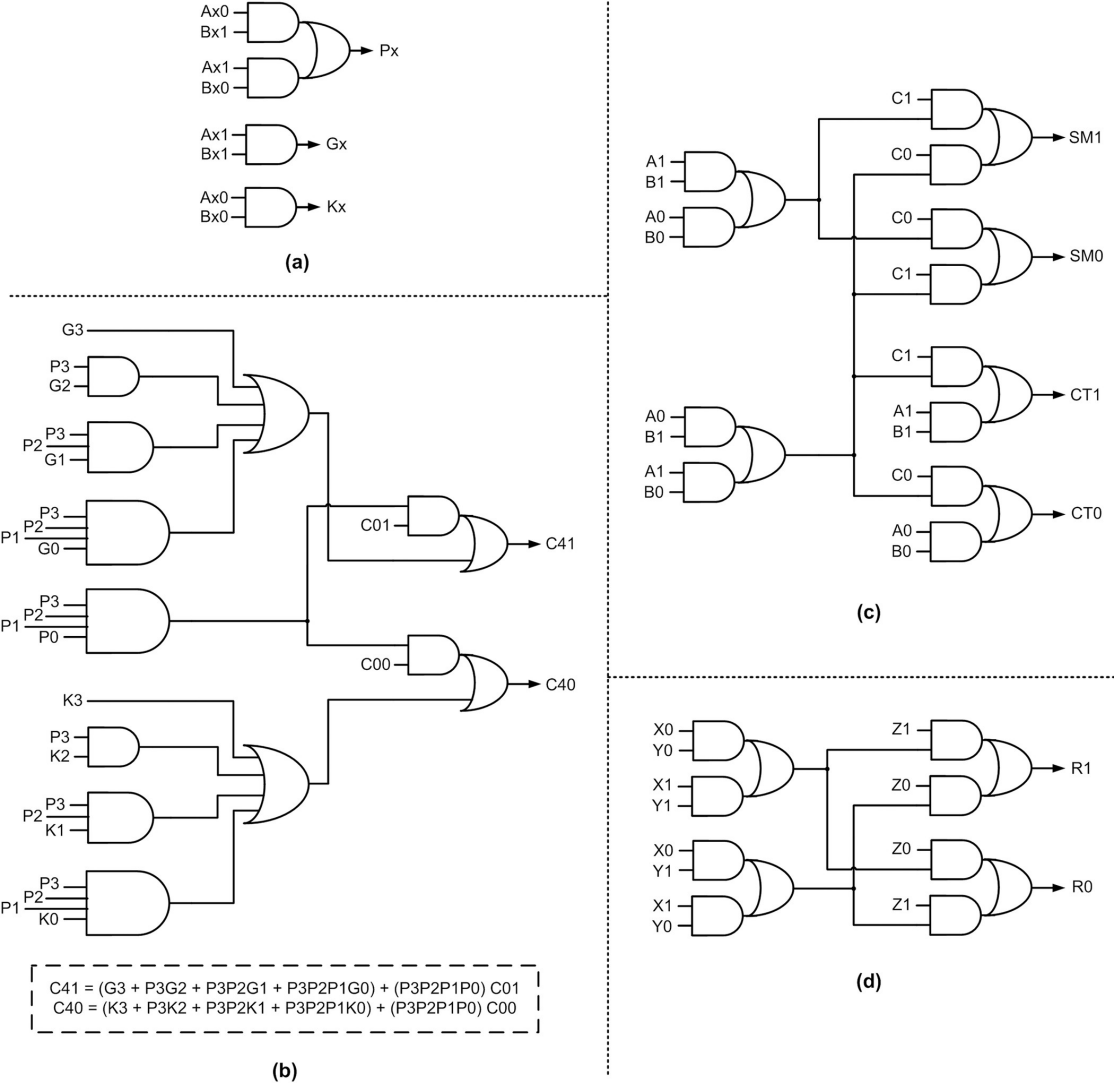
Building blocks of proposed monotonic asynchronous BCLA employing dual-rail encoding and corresponding to RZH: (a) generic realization of carry propagate, generate and kill functions; (b) example 4-bit BCLG implementation; (c) monotonic full adder; and (d) monotonic 3-input XOR function.

Fig 6 shows the constituents of the proposed monotonic 4-bit BCLA module corresponding to RZH, which can be replicated and cascaded to realize an N-bit BCLA as shown in Fig 3(B). Nevertheless, any BCLA module of size M-bits can be realized by taking a cue from Fig 6. To obtain the equivalent circuit of the monotonic 4-bit BCLA module corresponding to ROH, all the gates shown in Fig 6 should be replaced by their duals, i.e., AND, OR, AO21, and AO22 gates in Fig 6 should be replaced by OR, AND, OA21, and OA22 gates to obtain the equivalent circuit corresponding to ROH.

Fig 6(A) shows the generic realization of carry propagate, carry generate, and carry kill functions, Fig 6(B) shows the monotonic 4-bit BCLG, Fig 6(C) shows a monotonic full adder, and Fig 6(D) shows the monotonic realization of a 3-input XOR function whose inputs are (X1, X0), (Y1, Y0) and (Z1, Z0) and the output is (R1, R0). An M-bit monotonic BCLG along with an M-bit monotonic RCA forms an M-bit monotonic BCLA module that can be replicated and cascaded to realize an N-bit monotonic BCLA. Three copies of the monotonic full adder shown in Fig 6(C) and one copy of the monotonic 3-input XOR function shown in Fig 6(D) can be combined to realize a monotonic 4-bit RCA that would form a part of a monotonic 4-bit BCLA module.

Based on Figs 3(B) and 6, the forward latency of the proposed N-bit BCLA would be given by O[D_BCLG_^4b_first^ + {(N/M)– 2}×D_BCLG_^4b_intermediate^ + D_RCA_^4b^], where D_BCLG_^4b_first^ denotes the propagation delay encountered in the first 4-bit BCLA module that processes inputs A(3) to A(0) and B(3) to B(0) in Fig 3(B). D_BCLG_^4b_intermediate^ denotes the propagation delay encountered in any subsequent 4-bit BCLA module up to the penultimate BCLA module. D_BCLG_^4b_first^ is distinguished from D_BCLG_^4b_intermediate^ in that the look-ahead carry C(4) output by the first 4-bit BCLA module is produced after traversing multiple levels of logic whereas the look-ahead carry output by successive 4-bit BCLA modules are produced after traversing one level of logic viz. an AO21 gate. The reverse latency of the monotonic N-bit BCLA would be an optimal O[D_BCLG_^4b^], which is the same as O[D_BCLG_^4b_first^]. This becomes possible because all the 4-bit BCLG modules and 4-bit RCAs comprising a monotonic N-bit BCLA can process and output the spacer in parallel regardless of the receipt of corresponding spacer carry inputs due to the monotonic logic realization. The cycle time of the monotonic N-bit BCLA is thus given by O[(D_BCLG_^4b_first^ ×2) + {(N/M)– 2}×D_BCLG_^4b_intermediate^ + D_RCA_^4b^], which is considerably less than the cycle times of N-bit QDI SCLA, BCLA, and BCLADC. The cycle time of the proposed SCLA and BCLA was also found to be less than the cycle time of asynchronous RCAs discussed in the previous section. This shall be substantiated by the design metrics presented in the next section. Table 1 gives a summary of the theoretical cycle time evaluated for various IO-mode asynchronous adders discussed previously for quick reference.

**Table 1:** Cycle time (theoretical) of N-bit IO-mode asynchronous adders. N-bit RCAs were constructed using N full adders. N-bit CLAs were constructed using M-bit CLA modules where N and M are even and N modulo M equals 0; here M = 4.

Asynchronous adder reference	Adder architecture	Cycle time (Approximate theoretical estimate)
[23‒25]	RCA ^a^	O[2×N×D_FA_]
[25, 27]	RCA ^b^	O[2×N×D_FA_]
[28, 29]	RCA ^b^	O[(N+2) ×D_FA_]
[30]	RCA ^b^	O[2×N×D_FA_]
	RCA ^b^	O[(N+2) ×D_FA_]
	RCA ^b^	O[(N+2) ×D_FA_]
[31]	RCA ^c^	O[(N+2) ×D_FA_]
[32]	RCA ^d^	O[(N+1) ×D_FA_]
	RCA ^e^	O[(N+1) ×D_FA_]
[34]	SCLA ^f^	O [{D_SCLA_^4b_first^ + ((N/M)– 1)×D_SCLA_^4b^)} ×2]
[36‒38]	BCLA ^f^	O[{D_BCLG_^4b_first^ + ((N/M)– 2)×D_BCLG_^4b_intermediate^)} ×2 + D_RCA_^4b^ + D_FA_]
	BCLADC ^f^	O[2×D_BCLGDC_^4b_first^ + {(N/M)– 1}×D_BCLGDC_^4b_intermediate^ + D_RCA_^4b^ + D_FA_]
Proposed	SCLA ^g^	[(2×D_SCLA_^4b_first^) + {(N/M)– 2}×D_SCLA_^4b_intermediate^ + D_SCLA_^4b_last^]
	BCLA ^g^	O[(2×D_BCLG_^4b_first^) + {(N/M)– 2}×D_BCLG_^4b_intermediate^ + D_RCA_^4b^]

^a^ Full adder used to construct this RCA is strongly indicating.

^b^ Full adder used to construct this RCA is weakly indicating.

^c^ Full adder used to construct this RCA is of early-output type and QDI.

^d^ AOPT_EO_FA early output type full adder used to construct this RCA, which is relative-timed.

^d^ LOPT_EO_FA early output type full adder used to construct this RCA, which is relative-timed.

^f^ Constituent SCLA, BCLA, and BCLADC modules are of early-output QDI type.

^g^ Constituent SCLA and BCLA modules are of early output type and monotonic (non-QDI) realizations.

## 5. Design metrics

We considered 32-bit addition as an example and realized it using the asynchronous RCAs and CLAs discussed. A typical IO-mode asynchronous circuit stage comprising an input register bank and the asynchronous circuit (here, adder), as shown in Fig 1 was implemented. The acknowledgment input signal (AKI) was assumed to be supplied from the environment. The adders were realized in a semi-custom design style using a 28-nm CMOS standard digital cell library [43]. The cell library does not comprise a native C-element and so this was custom designed to realize the registers and completion detect logic. The proposed CLAs (SCLA and BCLA), being monotonic, do not embed the C-element in their logic whereas the existing QDI CLAs incorporate the C-element in their logic realization. The C-element was also used to realize the logic of QDI RCAs and CLAs discussed in Section 3.

A typical case high V_t_ library specification [43] was considered for simulation and synthesis, using a recommended supply voltage of 1.05V and an operating temperature of 25°C. Synopsys tools were used to simulate and estimate the design metrics of asynchronous adders. About a thousand randomly generated inputs were supplied through a test bench to the asynchronous adders at a constant latency of 15ns (to accommodate the slowest adder) to simulate and verify their functionality. The switching activity was recorded, which was subsequently used to estimate the total power dissipation. Two test benches were used, one corresponding to RZH and another corresponding to ROH, but both are logically equivalent. This helps to distinguish the variation in power based on RZH and ROH besides performing the functional simulation. Default wire loads were included in the estimation of design metrics and a fanout-of-4 drive strength was uniformly assigned to all the output ports i.e., the sum bits of the adders. Following an advanced timing analysis, a virtual clock was used to constrain the input and output ports of the adders. However, the clock being virtual does not form a part of the implementation. The forward latency of the asynchronous adders (which is equivalent to the critical path delay of synchronous adders) was estimated directly while the reverse latency of the asynchronous adders was estimated based on the delay given in the timing reports, as done in [37, 38]. Subsequently, the cycle time was calculated as the sum of forward and reverse latencies, which signifies the time taken to complete a data transaction.

The standard design metrics estimated for the asynchronous adders corresponding to RZH and ROH are given in Tables 2 and 3 respectively. The input register bank and the completion detect logic are the same for all the adders; only the underlying logic differs between the adders. Hence, the differences between the design metrics of adders given in Tables 2 and 3 are attributed to the differences between the adder logic. Adder legends are used in Tables 2 and 3 for ease of referencing while discussing the results and plotting the energy of the asynchronous adders.

**Table 2:** Design metrics of different 32-bit asynchronous adders corresponding to RZH, implemented using a 28-nm CMOS process.

Reference	Adder architecture	Adder legend	Timing parameters			Area (μm^2^)	Power (μW)
			FL ^α^ (ns)	RL ^β^ (ns)	CT ^γ^ (ns)		
[23]	RCA ^a^	AZ1	14.70	14.70	29.40	2518.32	1446
[24]	RCA ^a^	AZ2	9.12	9.12	18.24	2282.47	1429
[25]	RCA ^a^	AZ3	9.34	9.34	18.68	2493.93	1449
	RCA ^b^	AZ4	8.31	8.31	16.62	2412.60	1445
[27]	RCA ^b^	AZ5	7.07	7.07	14.14	2005.96	1415
[28]	RCA ^b^	AZ6	4.52	0.74	5.26	2087.28	1431
[29]	RCA ^b^	AZ7	3.40	0.82	4.22	2038.49	1421
[30]	RCA ^b^^,1^	AZ8	8.97	8.97	17.94	2103.55	1424
	RCA ^b^^,2^	AZ9	6.20	1.04	7.24	2339.40	1437
	RCA ^b^^,3^	AZ10	6.64	1.42	8.06	2282.47	1451
[31]	RCA ^c^	AZ11	3.19	0.70	3.89	1648.12	1405
[32]	RCA ^d^	AZ12	3.14	0.73	3.87	1534.27	1396
	RCA ^e^	AZ13	3.02	0.72	3.74	1648.12	1403
[34]	SCLA^f^	AZ14	2.84	2.84	5.68	2558.98	1469
[36]	BCLA ^f^	AZ15	3.22	2.98	6.20	2514.25	1443
	BCLADC ^f^	AZ16	2.40	1.77	4.17	2549.83	1450
[37]	BCLA ^f^	AZ17	2.84	2.59	5.43	2199.11	1437
	BCLADC ^f^	AZ18	2.09	1.45	3.54	2234.69	1443
[38]	BCLA ^f^	AZ19	3.55	3.29	6.84	2296.70	1454
	BCLADC ^f^	AZ20	1.85	1.19	3.04	2332.28	1460
Proposed	SCLA ^g^	PSZ	1.47	0.62	2.09	1739.62	1418
	BCLA ^g^	PBZ	1.47	0.58	2.05	1656.26	1397

^α^ FL–Forward latency

^β^ RL–Reverse latency

^γ^ CT–Cycle time.

^a^ Full adder used in this RCA is strongly indicating.

^b^ Full adder used in this RCA is weakly indicating; ^1^ SN full adder; ^2^ SNFC full adder; ^3^ SNX full adder

^c^ Full adder used in this RCA is of early-output type and QDI.

^d^ AOPT_EO_FA early output type full adder is used to construct this RCA, which is relative-timed.

^e^ LOPT_EO_FA early output type full adder is used to construct this RCA, which is relative-timed.

^f^ Constituent SCLA, BCLA, and BCLADC modules are of early-output QDI type.

^g^ Constituent SCLA and BCLA modules are of early output type and monotonic non-QDI realizations.

**Table 3:** Design metrics of different 32-bit asynchronous adders corresponding to ROH, implemented using a 28-nm CMOS process.

Reference	Adder architecture	Adder legend	Timing parameters			Area (μm^2^)	Power (μW)
			FL ^α^ (ns)	RL ^β^ (ns)	CT ^γ^ (ns)		
[23]	RCA ^a^	AO1	14.24	14.24	28.48	2518.32	1445
[24]	RCA ^a^	AO2	8.97	8.97	17.94	2282.47	1429
[25]	RCA ^a^	AO3	8.84	8.84	17.68	2363.80	1443
	RCA ^b^	AO4	8.12	8.12	16.24	2347.53	1442
[27]	RCA ^b^	AO5	7.04	7.04	14.08	2005.96	1415
[28]	RCA ^b^	AO6	3.88	0.73	4.61	2087.28	1431
[29]	RCA ^b^	AO7	3.39	0.81	4.20	2038.49	1421
[30]	RCA ^b^^,1^	AO8	9.05	9.05	18.10	2103.55	1424
	RCA ^b^^,2^	AO9	6.31	1.03	7.34	2339.40	1437
	RCA ^b^^,3^	AO10	6.75	1.19	7.94	2282.47	1456
[31]	RCA ^c^	AO11	3.02	0.70	3.72	1648.12	1404
[32]	RCA ^d^	AO12	3.16	0.72	3.88	1534.27	1395
	RCA ^e^	AO13	2.99	0.70	3.69	1648.12	1402
[34]	SCLA ^f^	AO14	2.82	2.82	5.64	2558.98	1469
[36]	BCLA ^f^	AO15	3.15	2.92	6.07	2546.78	1442
	BCLADC ^f^	AO16	2.34	1.75	4.09	2582.36	1449
[37]	BCLA ^f^	AO17	2.84	2.59	5.43	2199.11	1437
	BCLADC ^f^	AO18	2.04	1.45	3.49	2218.42	1442
[38]	BCLA ^f^	AO19	3.47	3.22	6.69	2304.83	1453
	BCLADC ^f^	AO20	1.83	1.23	3.06	2340.41	1459
Proposed	SCLA ^g^	PSO	1.45	0.65	2.10	1747.75	1418
	BCLA ^g^	PBO	1.48	0.62	2.10	1664.39	1395

^α^ FL–Forward latency

^β^ RL–Reverse latency

^γ^ CT–Cycle time.

^a^ Full adder used in this RCA is strongly indicating.

^b^ Full adder used in this RCA is weakly indicating; ^1^ SN full adder; ^2^ SNFC full adder; ^3^ SNX full adder

^c^ Full adder used in this RCA is of early-output type and QDI.

^d^ AOPT_EO_FA early output type full adder is used to construct this RCA, which is relative-timed.

^e^ LOPT_EO_FA early output type full adder is used to construct this RCA, which is relative-timed.

^f^ Constituent SCLA, BCLA, and BCLADC modules are of early-output QDI type.

^g^ Constituent SCLA and BCLA modules are of early output type and monotonic non-QDI realizations.

Tables 2 and 3 reflect almost a similar trend in the design metrics of different adders, and the practical cycle time estimates correlate well with the theoretical cycle time prediction given in Table 1. RCAs constructed using strong-indication full adders [23‒25] not only have the same forward and reverse latencies but the latency of such full adders is also high. This explains why AZ1, AZ2, and AZ3 have greater cycle times in Table 2 and AO1, AO2, and AO3 have greater cycle times in Table 3. As noted in Table 1, some of the RCAs incorporating weak-indication full adders have a cycle time of O[N×D_FA_] while the others have a cycle time of O[(N+2) ×D_FA_]. Thus, AZ4, AZ5, and AZ8 in Table 2 and AO4, AO5, and AO8 in Table 3 feature a cycle time with equal forward and reverse latencies while AZ6, AZ7, AZ9, and AZ10 in Table 2 and AO6, AO7, AO9, and AO10 in Table 3 feature a cycle time with much reduced reverse latency. RCAs incorporating early output full adders would process the spacer quickly compared to the processing of data, and the data may be processed slightly faster than in RCAs incorporating weak-indication full adders. Hence, AZ11, AZ12, and AZ13 in Table 2 and AO11, AO12, and AO13 in Table 3 have reduced cycle time than the rest of the RCAs.

The SCLA architecture would help to reduce the forward latency of an asynchronous adder. However, the SCLA comprising indicating or early output QDI modules would consume the same time for processing the spacer as the data and so its forward and reverse latencies are equal, which causes an increase in the cycle time. AZ14 in Table 2 and AO14 in Table 3 have a cycle time that is greater than the cycle time of some of the RCAs (AZ6, AZ7, AZ11, AZ12, AZ13 in Table 2, and AO6, AO7, AO11, AO12, AO13 in Table 3). This is because these RCAs have a much-reduced reverse latency (compared to their forward latency), which could not be achieved in an SCLA.

As described in Section 3, and highlighted in Table 1, the BCLA architecture realized using indicating or early output QDI modules is comparable to the SCLA architecture in terms of the cycle time although its reverse latency is moderately less than its forward latency. Hence, AZ15, AZ17, and AZ19 in Table 2, and AO15, AO17, and AO19 in Table 3 have cycle time that is comparable to the cycle time of the SCLA. As noted in Section 3, the BCLADC architecture was proposed specifically for IO-mode asynchronous design to improve the speed compared to SCLA and BCLA architectures comprising indicating or early output QDI modules. Thus, AZ16, AZ18, and AZ20 were found to have reduced cycle time than AZ14, AZ15, AZ17, and AZ19 in Table 2, and AO16, AO18, and AO20 were found to have reduced cycle time than AO14, AO15, AO17, and AO19 in Table 3.

The proposed CLAs (SCLA and BCLA) have two main advantages compared to their counterparts. Firstly, the proposed CLAs being monotonic and non-QDI requires less logic than CLAs which comprise indicating or early output QDI modules (such as QDI SCLA, BCLA, and BCLADC) and do not involve the C-element in their logic realization. The C-element has been used only for the registers and the completion detect logic. To make some comparisons, the area of the 4-bit SCLA module of [34] is 223.65 μm^2^ while the area of the proposed 4-bit SCLA module is 121.23 μm^2^ which implies a 45.8% reduction with respect to RZH. With respect to ROH, the proposed 4-bit SCLA module (122.24 μm^2^) has a 45.3% reduced area than the 4-bit SCLA module (221.61 μm^2^) of [34]. Among the QDI BCLAs [36–38], the BCLA design of [37] is found to be better. In comparison with the 4-bit BCLG module of [37], which consumes 73.96 μm^2^ of silicon for RZH and 71.92 μm^2^ of silicon for ROH, the proposed 4-bit BCLG module consumes 54.90 μm^2^ of silicon for RZH and 55.91 μm^2^ of silicon for ROH, thus achieving respective reductions in the area by 25.8% and 22.3%. Among the QDI BCLADCs [36–38], the design presented in [38] is found to be better. The 4-bit BCLGDC comprising the BCLADC [38] occupies an area of 91.24 μm^2^ for RZH and 92.25 μm^2^ for ROH. The 4-bit BCLG of [38] occupies an area of 86.15 μm^2^ for RZH and 87.17 μm^2^ for ROH. Compared to these, the proposed 4-bit BCLG occupies less area requiring 54.90 μm^2^ of silicon for RZH and 55.91 μm^2^ of silicon for ROH. Therefore, the reduced area occupancy of the proposed CLAs compared to the rest of the CLAs translates into a reduction in power dissipation, as evident from Tables 2 and 3.

Secondly, the proposed CLAs are constructed using CLA modules which require the least possible time for processing the spacer. This becomes possible since each CLA module of the proposed CLAs can process and produce the spacer as the output independently and simultaneously and this helps to achieve the least possible reverse latency. Given this, there does not arise a need to have a double carry logic and so the BCLADC architecture is not relevant to our proposition. The proposed CLAs realize the best of both worlds which are reducing the forward latency compared to an RCA through the provision of the look-ahead carry output logic and achieving a reduced reverse latency that is comparable to or better than an early output or relative-timed RCA by incorporating CLA modules that can process and produce the spacer faster. These two reasons explain why the proposed CLAs have less forward latency and reverse latency and thus lesser cycle time compared to the rest in Tables 2 and 3.

Among the proposed CLAs, the proposed BCLA corresponding to RZH (i.e., PBZ in Table 2) is found to be preferable as it has a slight edge over PSZ, PSO, and PBO overall. In terms of the cycle time, compared to the best of existing designs given in Table 2 (which is AZ20), PBZ reports a 32.6% reduction in cycle time, a 29% reduction in area, and a 4.4% reduction in power for RZH. Likewise, for ROH, PBO reports a 31.4% reduction in cycle time, a 28.9% reduction in area, and a 4.4% reduction in power. Nevertheless, PBZ is seen to be marginally superior to PBO from Tables 2 and 3.

**Fig 7:**
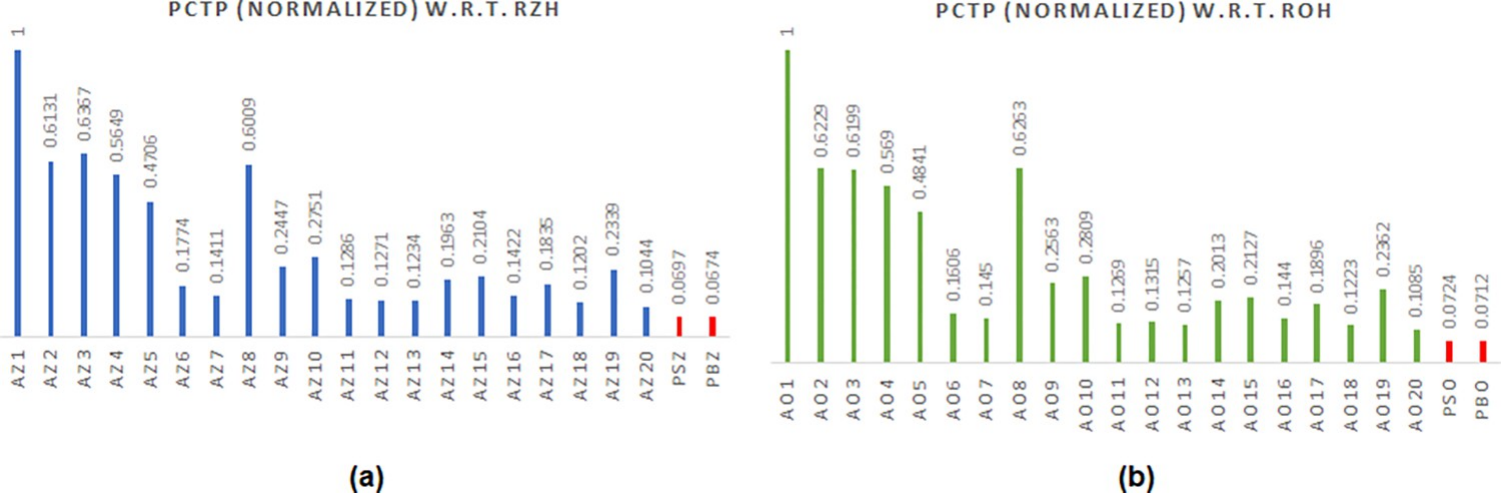
Normalized power-cycle time product (PCTP) of 32-bit asynchronous adders corresponding to (a) return-to-zero handshaking, and (b) return-to-one handshaking. Adder legends used in (a) and (b) are referred to in Tables 2 and 3 respectively. The normalized PCTP values of proposed CLAs are highlighted by the red bars in (a) and (b).

It would be useful to estimate the energy of asynchronous adders, which is a widely regarded figure of merit for low-power design [44]. For a synchronous circuit, energy is given by the power-delay product (PDP) where power and delay are preferred to be less, and hence PDP is also preferred to be less. For an (IO-mode) asynchronous circuit, energy is specified by the power-cycle time product (PCTP). Given that power and cycle time are preferred to be less, therefore PCTP is also preferred to be less. In other words, an asynchronous adder having the least PCTP is an energy-efficient design. Based on the estimated design metrics (given in Tables 2 and 3), the PCTP of all the asynchronous adders was calculated corresponding to RZH and ROH separately. Then, the PCTP was normalized. To do the normalization, the highest PCTP value was considered as the baseline and this was used to divide the actual PCTP of all the asynchronous adders. This kind of normalization was done for RZH and ROH separately. The normalized PCTP plots of asynchronous adders corresponding to RZH and ROH are portrayed in Fig 7(A) and 7(B) respectively, where the red bars highlight the normalized PCTP values of the proposed CLAs viz. PSZ and PBZ correspond to RZH, and PSO and PBO correspond to ROH. From Fig 7(A) and 7(B), it is seen that the proposed asynchronous CLAs achieve superior energy efficiency compared to their counterparts, and PBZ has a slight edge over PSZ, PSO, and PBO in terms of energy, and hence it is preferred.

## 6. Conclusions

With respect to IO-mode asynchronous circuits, QDI asynchronous circuits guarantee delay insensitivity internally and externally but their design metrics are generally expensive. In comparison, non-QDI asynchronous circuits such as relative-timed circuits and monotonic circuits are simpler and relaxed and could facilitate improved performance metrics. Relative-timed circuits tend to incorporate sophisticated timing assumptions to sequence the arrival of inputs to process and produce the outputs. In comparison, monotonic circuits are less sophisticated in that they guarantee delay insensitivity externally and ensure the monotonicity of signal transitions between the primary inputs and outputs. Although QDI circuits are more robust, their successful operation is subject to the satisfying of isochronic fork assumptions imposed internally within the circuit, and a violation of those would affect the delay insensitivity. Given this, in practice, monotonic circuits tend to operate similarly to QDI circuits. Therefore, concerning asynchronous logic design, monotonic circuits are a practically viable alternative to QDI circuits, but this category of asynchronous circuits has been sparingly explored in the literature. Given this, to our knowledge, this paper has presented the first generic designs of monotonic asynchronous adders viz. a monotonic SCLA and a monotonic BCLA, which report superior performance metrics compared to QDI asynchronous adders.

Among the proposed CLAs, the monotonic BCLA corresponding to RZH is noted to have a slight edge over other monotonic CLAs. Compared to the best of the existing QDI asynchronous adders (BCLADC) in the literature, determined based on cycle time, the proposed BCLA achieves a 32.6% reduction in cycle time, a 29% reduction in area, a 4.3% reduction in power, and a 35.5% reduction in energy for RZH, and (ii) a 31.4% reduction in cycle time, a 28.9% reduction in area, a 4.4% reduction in power, and a 34.4% reduction in energy for ROH. Given the significant improvements in design metrics achieved by the proposed asynchronous adder, our future work would consider the monotonic design of other practically useful arithmetic circuits such as multipliers, dividers, etc.
